# PolyaPeak: Detecting Transcription Factor Binding Sites from ChIP-seq Using Peak Shape Information

**DOI:** 10.1371/journal.pone.0089694

**Published:** 2014-03-07

**Authors:** Hao Wu, Hongkai Ji

**Affiliations:** 1 Department of Biostatistics and Bioinformatics, Emory University, Atlanta, Georgia, United States of America; 2 Department of Biostatistics, Johns Hopkins University, Baltimore, Maryland, United States of America; University of California, Los Angeles, United States of America

## Abstract

ChIP-seq is a powerful technology for detecting genomic regions where a protein of interest interacts with DNA. ChIP-seq data for mapping transcription factor binding sites (TFBSs) have a characteristic pattern: around each binding site, sequence reads aligned to the forward and reverse strands of the reference genome form two separate peaks shifted away from each other, and the true binding site is located in between these two peaks. While it has been shown previously that the accuracy and resolution of binding site detection can be improved by modeling the pattern, efficient methods are unavailable to fully utilize that information in TFBS detection procedure.

We present PolyaPeak, a new method to improve TFBS detection by incorporating the peak shape information. PolyaPeak describes peak shapes using a flexible Pólya model. The shapes are automatically learnt from the data using Minorization-Maximization (MM) algorithm, then integrated with the read count information via a hierarchical model to distinguish true binding sites from background noises.

Extensive real data analyses show that PolyaPeak is capable of robustly improving TFBS detection compared with existing methods. An R package is freely available.

## Introduction

One major goal of functional genomics is to comprehensively characterize the regulatory circuitry behind coordinated spatial and temporal gene activities. In order to achieve this goal, a critical step is to monitor downstream regulatory programs of various transcription factors (TFs). ChIP-seq [1], [2], a technology that couples chromatin immunoprecipitation with massively parallel sequencing, is capable of mapping genome-wide transcription factor binding sites (TFBSs), and is increasingly used by scientists and nation-wide projects such as ENCODE [3] and modENCODE [4] to annotate functional sequence elements in human genome and genomes of model organisms. ChIP-seq data grow rapidly. The first ChIP-seq studies were published in 2007. Since then, several thousands studies have been performed and data are available in public databases [5]. This highlights the importance of continuous development of robust and powerful ChIP-seq data analysis tools.

The raw data produced by a ChIP-seq experiment are tens of millions of short (usually less than 100 base pairs) DNA sequences called “reads”. To identify TFBSs, the reads are first aligned to a reference genome and the uniquely aligned reads are retained. Next, the genome is scanned to identify “peaks”, or regions enriched in aligned sequence reads, which are the predicted TF binding sites. Since 2007, a number of peak calling algorithms and software tools have been developed. Examples include BayesPeak [6], CisGenome [7], FindPeaks [8], GPS [9], Hpeak [10], MACS [11], MOSAiCS [12], PeakSeq [13], PICS [14], QuEST [15], SISSRs [16], T-PIC [17], etc. Several benchmark studies have also been conducted to compare different peak calling tools [18], [19].

**Figure 1:**
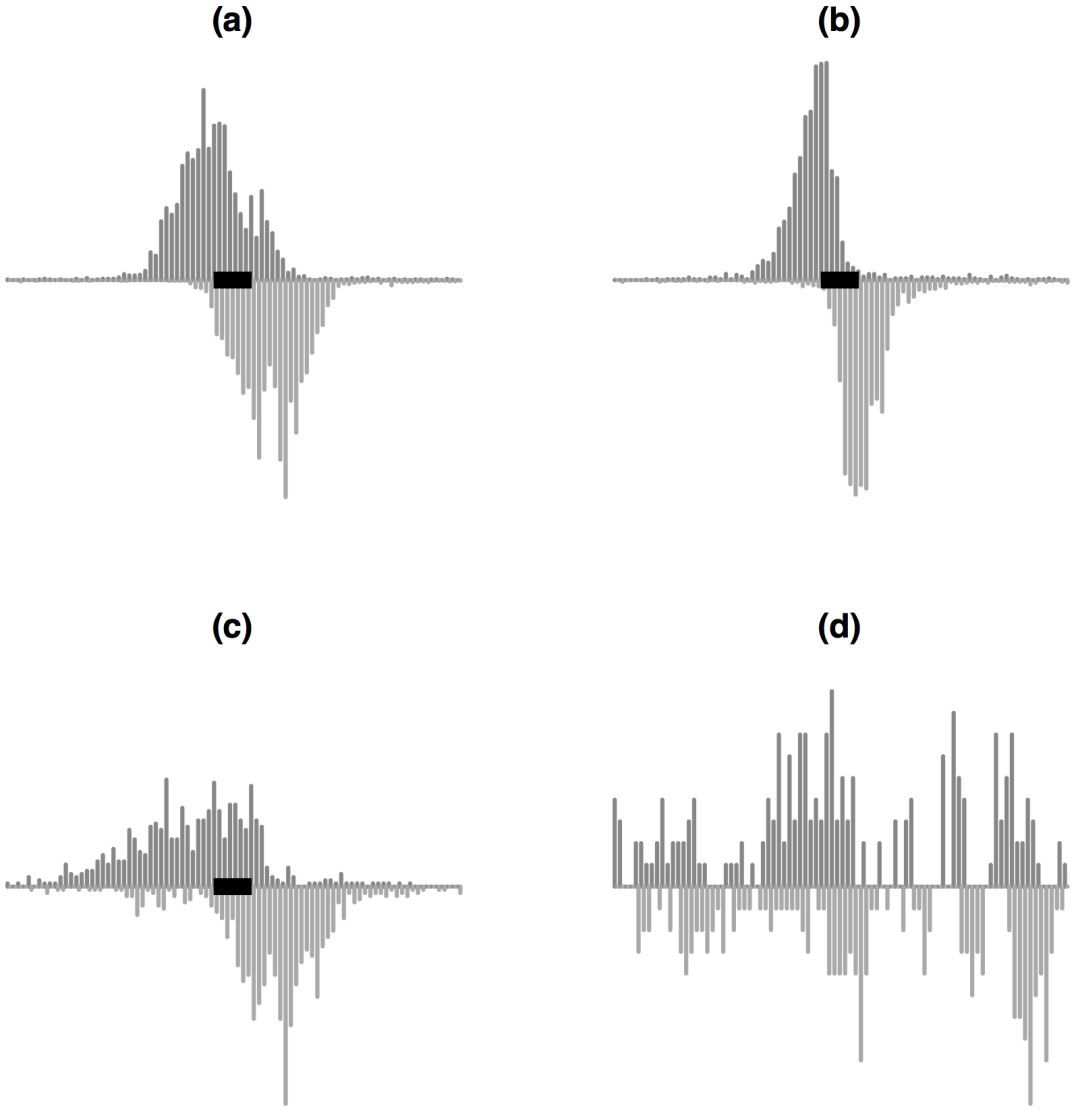
Illustration of peak shapes from three good binding sites (a, b, c) and one false positive (d). X-axis is genomic location centered at the summit of each peak. Y-axis represents ChIP read counts in 10 bp genomic windows. For each peak, data from the IP sample in a 800 bp window surrounding the MACS peak summit is shown. Bars above the middle represent counts from forward strand, and bars below represent the counts from reverse strand. The black rectangles illustrate the TF binding sites. (a) and (b) are two different binding sites from the same ChIP-seq dataset, and (c) is a binding site from a different dataset. They illustrate that peak shapes at true TFBSs are similar but can vary across binding sites and datasets.

Early analyses revealed that ChIP-seq data for mapping TFBSs have a characteristic pattern: surrounding each true binding site, sequence reads aligned to the forward and reverse strands of the reference genome are clustered into two distinct peaks that are shifted away from each other, and the binding site is located in between them (Figure 1). This phenomenon is caused by the sequencing protocol which involves cutting chromatin into fragments and reading the sequences from both ends of the TF bound DNA fragments. The cutting points seldom fall within the binding sites since the DNA is protected by the TF. As a result, in most cases the binding sites sit within the DNA fragments, and their flanking sequences are read out by the sequencer. Since the machine reads the DNA sequences in a directional way, reads from one end of the DNA fragments are always aligned to the forward strand of the reference genome, and reads from the other end are always aligned to the reverse strand. This creates the bimodal peak pattern shown in Figure 1.

This pattern has been shown to be useful for improving TFBS detection. For example, SISSRs [16] uses the sign change of the forward and reverse strand read count difference along the genome to identify the true binding sites. CisGenome [7] uses the summits of the two coupled peaks to determine the boundaries of binding sites. QuEST [15] and MACS [11] estimate the offset between the forward and reverse strand peaks, and shift these two peaks together based on the offset. They then merge signals from both peaks to increase accuracy for identifying true binding location. While many state-of-the-art peak callers use the bimodal pattern in their design, most methods only use the information contained in the offset between the two coupled peaks. Few method fully utilize the information contained in the peak shapes. As pointed out in a recent publication [20], many false positive TFBSs can be “filtered out by visual inspection on the peak sizes and appearance”. To demonstrate, Figure 1 shows three examples of good binding sites along with an example of false positive. All examples have large read counts in both strands with certain offsets. Methods using the offset information alone would call all examples as binding sites. However, the example in Figure 1(d) is very likely to be sequencing artifacts. It does not contain DNA motifs for the TF, and clearly has very different shapes from the true binding sites. Thus if the peak shape information is used in the inference, this false positive can be eliminated.

There are two existing methods fully incorporate the peak shape information in a rigorous statistical framework. PICS [14] uses two t-distributions with shifted centers to jointly model the positions of forward and reverse reads. A limitation of this approach is that it assumes that the peak shapes can be described by scaled t-distributions, which may not reflect the true peak shapes as they could be asymmetric. GPS [9] implements a more flexible approach to use an empirical distribution to characterize the peak shapes. The estimation of the shapes and peak calling are iterated until convergence. Both PICS and GPS are computationally intensive, especially when the total read counts is large because they model the position of all aligned reads. T-PIC [17] is another method that partially uses the peak shape information by summarizing it into a one-number statistic. However it only reflects certain aspects of the peak shape and cannot capture the full detail. Moreover, T-PIC does not treat the forward and reverse strand reads separately and ignores the offset between them.

In this article we propose a new method called PolyaPeak to utilize the peak shape information for detecting TFBSs. PolyaPeak models the read counts from equal sized bins around the binding sites and describes the peak shapes using a multivariate Pólya distribution. It then uses a hierarchical model to integrate the peak shape and the read count information to identify binding sites. Compared with PICS and GPS, PolyaPeak models the bin counts so it's more computationally efficient and can be easily embedded into MACS or CisGenome as a downstream peak ranking algorithm. Therefore, PolyaPeak provides a more flexible and efficient model for utilizing the peak shape information. Our extensive real data analyses show that its performance is robustly among the bests compare to several state-of-the-art peak callers.

## Materials and Methods

We use a two-step procedure to detect binding sites. In the first step, a simple and fast peak calling algorithm based on smoothing is applied to roughly identify the locations and summits of candidate peaks. In this article we use MACS as the first step peak caller although one can easily replace MACS by other methods. In the second step, the candidate peaks are scored and ranked by a more sophisticated model which considers both the read count and the peak shape information. Since the first step is based on existing algorithms, this article focuses on the second step.

### A hierarchical model for peak scoring

Assume there are 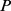 candidate peaks obtained from the first step peak calling. For each peak, we take an 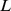 (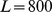 by default) base pair (bp) window centered at its summit. The window is divided into equal sized non-overlapping bins of 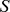 bp long (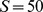 by default). The number of reads within each bin is obtained. Reads aligned to the forward and reverse strands are counted separately. Hereinafter, “peak” refers to the 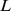 bp window. For peak 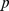, denote the read counts on the forward and reverse strands in bin 0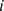 and sample 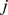 by 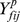 and 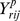 respectively. Here 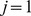 or 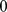 refers to IP or control sample, and 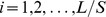. Reads from replicate samples are pooled together. Define 
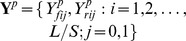
 to be read counts from all bins and all samples for peak 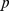. Let 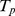 be the total read count in peak 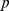: 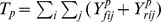.

Let 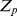 indicate whether peak 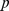 is a true binding site ( = 1) or not ( = 0). We assume that *a priori*, the probability for a candidate peak being a true binding site is 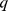, or 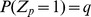. Then the observed bin-level read counts 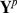 for each peak are assumed to be generated hierarchically. First, a total read count 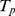 is drawn from certain distribution. Second, the 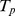 reads are randomly allocated to different bins based on a probability distribution that specifies the peak shape.

The distributions for generating 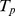 conditioned on 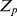 are:
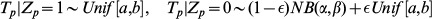
(1)In other words, if 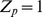, the candidate peak 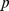 is a true binding site and 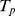 is assumed to follow a uniform distribution. If 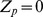, the candidate peak 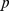 represents background noise and 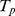 is assumed to follow a mixture of a negative binomial distribution and a uniform distribution. Negative binomial distribution is a popular choice for modeling the background read counts [7], [12]. Compared with Poisson, it allows over-dispersion and provides better fit to the data. Our choice of using a mixture of negative binomial and uniform for background is motivated by real data observation. It implies that for most background regions, the total counts follow a negative binomial distribution. However, some non-binding regions may have unusually large read counts due to artifacts. These outliers are modeled by the uniform mixing component. Technically, these model assumptions guarantee that the likelihood ratio 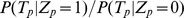 increases monotonically with 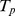, but is bounded at 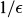 in interval 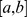. Thus the inference will not be overly influenced by the outliers, and the results will be more robust. In PolyaPeak, we set 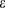 to 0.001 and treat it as fixed and known.

Given 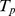, the bin counts 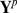 are assumed to follow multinomial distributions with random bin level probabilities 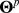: 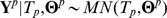. Here 
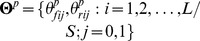
, and 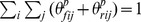. 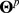 characterizes the peak shape at peak 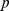. We further assume that the peak-specific multinomial probabilities 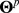 follow Dirichlet distributions with different parameters at background and binding regions:
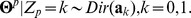
Here 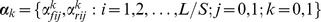. Conceptually, at a true binding site the proportions of multinomial distribution, e.g., 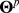, describe the peak shape. Using a Dirichlet prior for 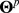 is based on observation that peaks in real data vary in widths, heights, and shapes, possibly due to various biological and technical factors. The Dirichlet prior provides a flexible model to allow the heterogeneity and variation in the peak shapes.

Integrating out the peak specific multinomial probabilities 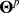, one can obtain the marginal bin counts distribution conditional on the total counts:
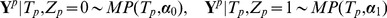
(2)Here MP represents multivariate Pólya distribution, also known as Dirichlet-multinomial compound distribution which generalizes the Beta-binomial distribution.

In the model above, the bin counts 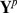 from all candidate peaks are the observed data. Unknown model parameters include 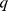, 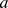, 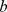, 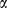, 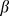, 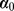 and 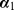. These parameters can be either specified or estimated from the data. Given the parameters, one can compute the posterior probability for a candidate peak being a true binding site. Such posterior probability can be decomposed into three components:
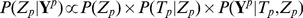
(3)The first component is the prior probability for peak 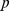 to be a true binding site. The second component is the information from the total read count. The third component is the allocation of read counts in different bins conditional on the total count. This component characterizes the peak shape around the binding site and was ignored by many existing peak callers. PolyaPeak first determines the unknown parameters, then compute 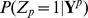 for each candidate peak and rank the candidate peaks accordingly.

### Choice of parameters

#### Choosing 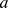, 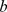, 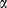 and 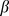


Given a list of candidate peaks, the distributional parameters in Equation 1 are determined as follows. For each candidate peak, we obtain the total read count 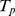. The minimum and maximum of 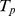 from all candidate peaks are taken as 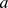 and 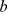 for the uniform distribution.

To estimate the negative binomial parameters 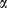 and 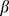, we first obtain genomic regions not covered by candidate peaks. These regions are cut into 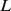 bp non-overlapping windows and read counts for each window are obtained. A large proportion of the genome (e.g., the repetitive regions) is unmappable. As a result, many background windows have zero count. In practice, if there are five consecutive regions with zero count, we exclude these regions from the analysis. The real data analyses show that the counts from the remaining regions can be fitted well by a negative binomial. Using these regions, we estimate 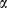 and 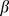 by a moment estimator. A negative binomial random variable 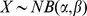 has 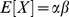 and 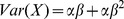. Let 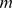 and 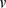 be the sample mean and variance for 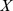, we get: 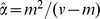 and 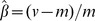.

#### Choosing 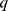


The first step peak calling algorithm provides a FDR estimate for the candidate peaks. We simply estimate 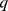 by 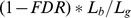. Here 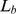 is the total length of the candidate peaks, and 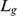 is the length of the genome after excluding the unmappable regions. Since the FDR estimate provided by the initial peak calling may be biased, the estimate of 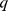 may be inaccurate. However, 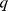 only affects the value of the posterior probability 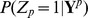. It will not change the final peak ranking provided by PolyaPeak. For this reason, we can tolerate the bias in estimating 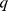, since it does not compromise our main goal of improving the peak ranking.

#### Estimating 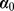 and 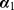


The procedures for estimating the parameters of multivariate Pólya distribution has been proposed previously. Here we implement the MM algorithm (reviewed in File S1) introduced by [21] to estimate 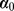 and 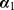.

For 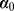, we first randomly sample 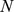 (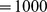 by default) genomic intervals of 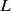 bps from the non-peak regions. Each interval is divided into equal sized bins of 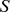 bps, and the bin read counts are obtained. Using these counts as input, 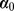 can be estimated via an MM algorithm. For 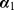, we first obtain 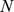 top ranked candidate peaks from the initial peak calling. These high-quality peaks are most likely to be true binding sites, and will be used as the training data to learn peak shapes. For each of the 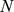 top peaks, we obtain the bin read counts 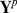. Using these bin counts as data, 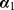 can be estimated using MM algorithm.

### Ranking the peaks

With all parameters determined, PolyaPeak will compute 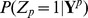, the posterior probability that each candidate peak is a true binding site, and use these posterior probabilities to rank the peaks. Some peaks may have the same posterior probabilities due to constraints of numerical precision. For these peaks, we use the log likelihood ratio 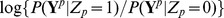 to break the ties.

### Implementation

The proposed method is implemented as an R package titled “PolyaPeak” for re-ranking a list of peaks reported from another peak calling software. The computational intensive part (MM algorithm) was implemented in C for efficiency. The software package can be freely downloaded from http://web1.sph.emory.edu/users/hwu30/polyaPeak.html.

## Results

### Data

We tested PolyaPeak on a large number of publicly available datasets and obtained similar results. Here we present representative results using six datasets generated by three different labs. The first two datasets were generated by [22] for mapping binding sites of mouse TFs OCT4 (POU5F1) and MYCN in mouse embryonic stem cells (mESC). The reads aligned to mouse genome build mm8 were downloaded from the Gene Expression Omnibus (GEO) (accession number GSE11431). The number of uniquely aligned reads for each sample ranged from 3 to 9 million. Each dataset contained one IP and one control sample. Datasets 3 and 4 were generated by Snyder lab at Yale/Stanford University for mapping the binding sites of MYC and MAX TFs in human K562 cell line. The data were generated as part of the ENCODE project. Sequence reads aligned to human genome build hg18 were downloaded from the ENCODE data coordination center. Each dataset contained two IP and one Input control samples. There were 10 to 20 million uniquely aligned reads in each sample. Datasets 5 and 6 were generated by the HudsonAlpha Institute, also for ENCODE project, for mapping the binding sites of GABP and NRSF TFs in human HepG2 cell line (GEO accession numbers GSM803343 for GABP and GSM803344 for NRSF). We downloaded the raw sequence reads aligned to human genome build hg19. Each dataset contained two IP and one Input control samples. Each sample had 50 to 100 million uniquely aligned sequence reads.

The diversity of the test data in terms of lab origin, cell type, species, reference genome and sequencing depth demonstrates the general applicability of PolyaPeak.

### Exploratory analysis

**Figure 2:**
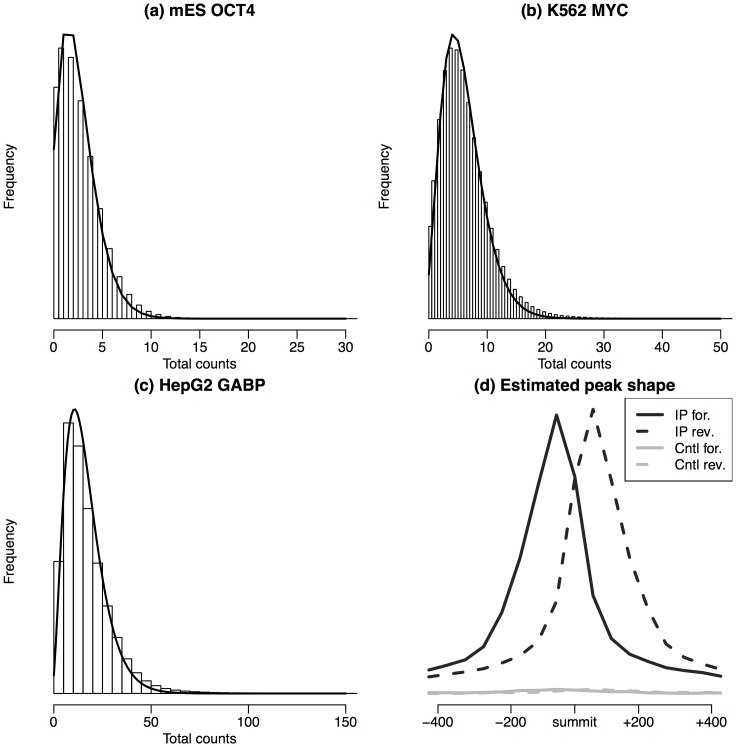
(a)–(c): histogram of total read counts in background regions from different datasets: (a) TF OCT4 in mouse ES cells; (b) TF MYC in K562 cell line; and (c) TF GABP in HepG2 cell line. The black solid curve is the theoretical density for the negative binomial distribution with parameters estimated from the method of moment procedure. It can be seen that the total counts in background regions can be approximated well by a negative binomial distribution, and the method of moments for estimating negative binomial parameters works well. (d) Estimated peak shape from polyaPeak for K562 MYC data.

First, we explored various characteristics of the real data. Figure 2(a)–(c) shows histograms of background window read counts 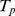 in the mESC OCT4, K562 MYC, and HepG2 GABP data. In each plot, the density curve of a negative binomial distribution with parameters estimated from the proposed method of moment estimator is also shown. It shows that the background total counts can be approximately modeled by a negative binomial distribution, and the estimation procedure for choosing negative binomial parameters works well.

Figure 2(d) shows the estimated peak shapes from the K562 MYC data. Plotted in the figure are the mean shapes for binding sites, e.g., 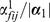 and 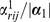 for 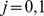. The estimated parameters are able to capture the location shift between the forward and reverse strand peaks, and the enrichment of IP compared with control samples. Figure 2 are only representative examples to illustrate key data characteristics. Analyses of the other datasets produced similar results.

### Comparison with other methods

Next, we compared PolyaPeak with several existing peak calling methods: MACS, CisGenome, PICS, GPS and T-PIC. While a number of peak calling methods have been developed, two comprehensive and independent benchmark studies showed that MACS robustly performs among the best in terms of peak calling sensitivity and overall receiver operating characteristics [18], [19]. For this reason, we used MACS as the baseline to benchmark our method. CisGenome is another popular software tool for peak calling with relatively high specificity. Currently, MACS and CisGenome are the two most cited ChIP-seq peak calling tools according to both the ISI Web of Science and Google Scholar using “ChIP-seq” as the query keyword. PICS, GPS and T-PIC are recently developed peak calling algorithms which attempt to use the peak shape information. This makes them different from other peak callers that do not fully utilize the shape information. Since the main point of this paper is to use peak shape to improve TFBS detection, we included PICS, GPS and T-PIC into our comparisons to test the effectiveness of our model.

For each dataset and each peak calling method except T-PIC, the analysis produced a ranked peak list. We compared different methods based on the enrichment of DNA motifs in the reported peaks. To avoid biases caused by peak lengths, we truncated or extended all peaks to make them having the same length (200 bps) before the motif analysis. For each ranked peak list, we evaluated the percentage of top 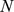 peaks containing at least one DNA motif site for the TF. We then plotted this percentage as a function of 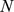. The DNA motif sites were obtained using the motif mapping function matchPWM in the Biostrings packages in BioConductor. The position weight matrices (PWMs) were obtained from TRANSFAC [23].

**Figure 3:**
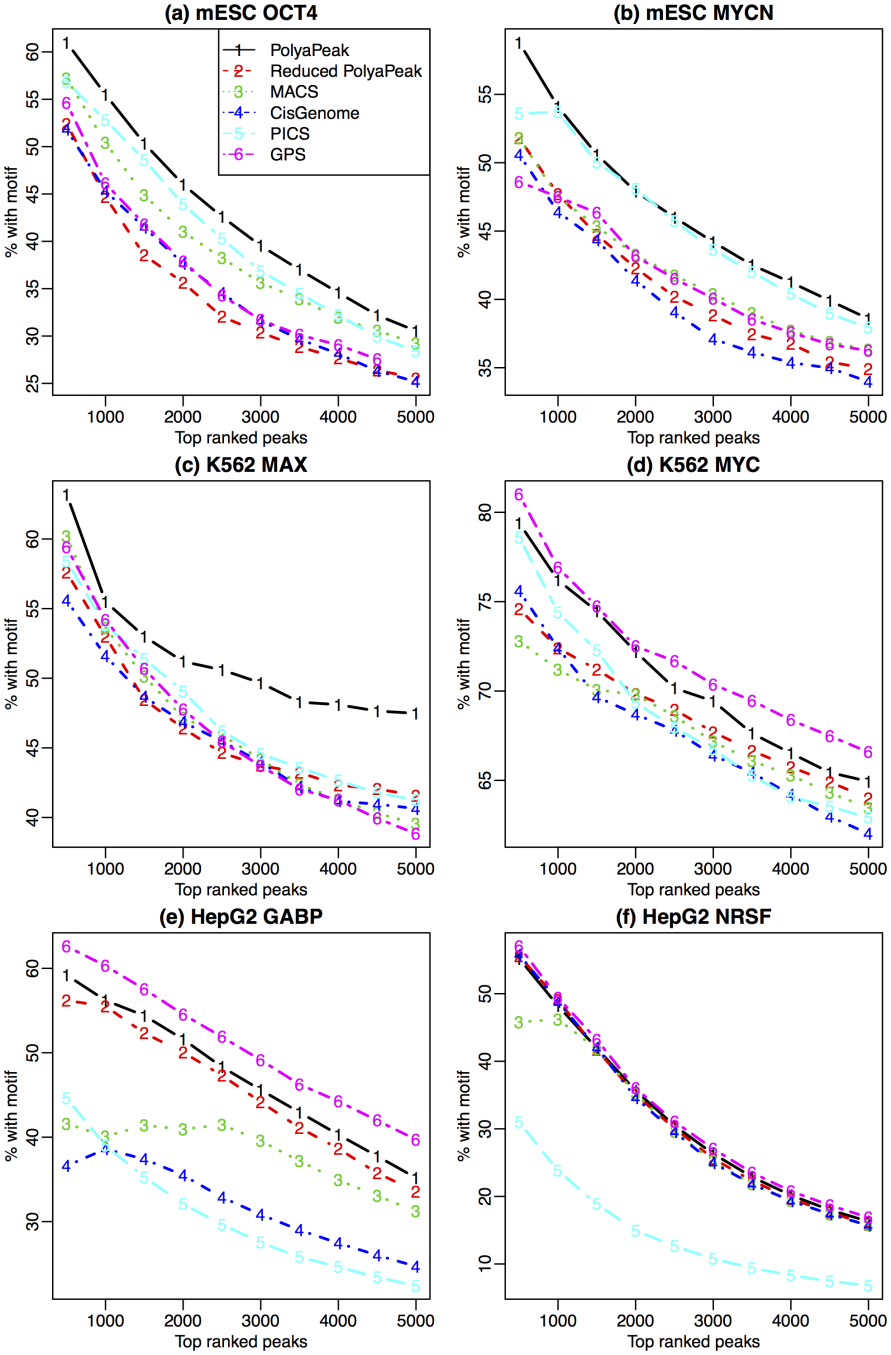
Comparison of motif enrichment in top ranked peaks reported from different methods (PolyaPeak, reduced PolyaPeak without peak shape information, MACS, CisGenome, PICS and GPS) for six public datasets. X-axis represents peak ranks. Y-axis represents the percentage of reported peaks containing at least one DNA motif site.

Figure 3 compares the motif contents in peaks ranked by different methods. The results show that top peaks reported by PolyaPeak consistently have higher or comparable motif enrichment level compared with peaks reported from MACS, CisGenome or PICS, and the improvement could be substantial. For example, in the HepG2 GABP data, 56% of of the top 1000 peaks reported by PolyaPeak contained at least one GABP motif, whereas the percentages was 40%, 38% and 40% for MACS, CisGenome and PICS respectively. Thus PolyaPeak improved MACS by 40%. Compared to GPS, PolyaPeak outperformed in mESC OCT4 and MYCN, as well as K562 MAX data. GPS outperformed PolyaPeak in K562 MYC and HepG2 GABP data. The two performed similarly in HepG2 NRSF data. These results showed that overall, the performance of PolyaPeak is slightly better than GPS in terms of motif enrichment. Among all existing methods, we found that overall GPS and PICS provides better results than MACS and CisGenome, which is not surprising since it has incorporated peak shape information. In all the data we have tested, PolyaPeak robustly performed among the bests.

The comparison to T-PIC was more difficult since T-PIC runs very slow and usually reports larger number of peaks without providing peak rankings. Therefore we were only able to roughly compare the performance based on the overall motif contents of all reported peaks. Details of this comparison are provided in File S1, and the results show that PolyaPeak again performed better than T-PIC.

### The net gain by modeling peak shapes

The observed differences between PolyaPeak and the other methods can be caused by many different factors, such as differences in statistical models, parameter estimation methods, or implementation details. MACS was used as the first step peak caller for PolyaPeak. Even for the relatively well-controlled comparison between PolyaPeak and MACS, the observed differences could be due to differences in (1) modeling the total read counts, i.e., 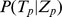, or (2) using the peak shape information, i.e., 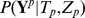. In order to determine whether the peak shape information really played an important role in improving the peak ranking performance, we developed a reduced PolyaPeak model by removing the peak shape information, and compared the reduced model with the original PolyaPeak that uses the peak shapes. To develop the reduced PolyaPeak model, we did not break peaks into 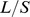 bins. Instead, for each peak we only counted the number of IP and control reads in the 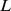 bp window centered at the peak summit. As a result, we obtained two numbers per peak: 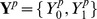 where 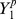 is the read count from the IP sample, and 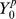 is the read count from the control sample. 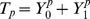 is the total read counts. We then modeled 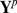 similarly as in Formulas 1–3. However, rather than having 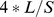 numbers per peak as the observed data, now we only have two numbers per peak. The reduced model still allows one to use the information from the IP and control read count differences, but the peak shape information is lost. We fit the reduced model and applied it to rank peaks in the same way as the original PolyaPeak. Figure 3 shows that the original PolyaPeak consistently outperformed the reduced model. This carefully controlled comparison clearly demonstrates that incorporating the peak shape information is able to substantially improve the accuracy for detecting true binding sites.

### The change of peak rankings

**Figure 4:**
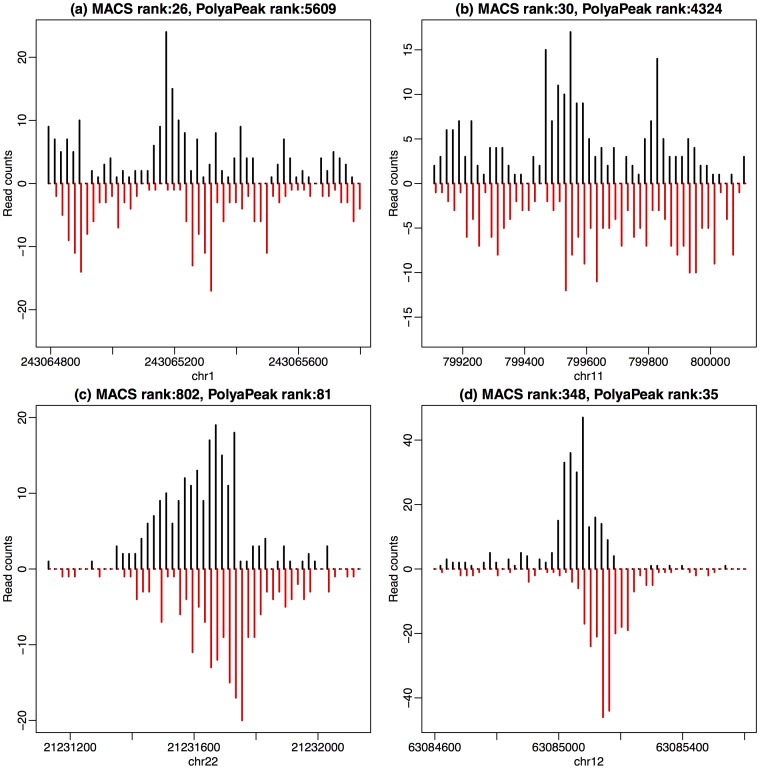
Examples of peaks showing large differences in ranks from MACS and PolyaPeak. (a) and (b): Peaks ranked high in MACS but low in PolyaPeak. (c) and (d): Peaks ranked low in MACS but high in PolyaPeak.

We further looked at the peaks with large rank changes between the results from MACS and PolyaPeak. Figure 4 shows several examples of MACS peaks from the K562 MYC data that are down- or up-ranked by PolyaPeak. Figure 4 (a) and (b) show two peaks with high ranks from MACS and low ranks from PolyaPeak. This type of peaks often contain large read counts but do not have a clear shape. None of the peaks shown here contained MYC motif sites. Therefore the high read counts in these regions very likely represent artifacts. Figure 4 (c) and (d) are two examples of peaks up-ranked by PolyaPeak (ranked low from MACS but high from PolyaPeak). Both of them have nice shapes, and contain MYC motif.

Furthermore, it is found that the peaks ranked high in MACS could have huge rank reduction in PolyaPeak. On the other hand, the top ranked peaks from PolyaPeak usually are also ranked high in MACS and seldom have dramatic rank reduction. These results suggest that PolyaPeak mainly worked as a false positive filter to down-rank low-quality MACS peaks. In general, MACS peaks down-ranked by PolyaPeak have lower motif content. As a result, the overall motif enrichment level is higher from PolyaPeak results, as shown in Figure 3.

## Discussion

Previous benchmark studies have shown that MACS robustly performs among the best compared to other peak callers. Our results show that by fully utilizing the peak shape information, PolyaPeak is able to robustly outperform MACS. Compared with the other two packages that uses peak shape information (PICS and GPS), PolyaPeak also outperforms based on our tests. PolyaPeak is computationally efficient because it models the read counts from equal sized bins, whereas both PICS and GPS model the positions of all aligned reads so their computational burden grows with total reads. While we deliver PolyaPeak as an R package, the computation intensive parts were written in C. On a computer with 2.6 GHz CPU and 32 GB RAM running Linux, the total computation time (using MACS to call peaks then PolyaPeak to rank peaks) for an experiment with 25 million total aligned reads is around 15 minutes. As a comparison, GPS takes over an hour and PICS takes more than two hours on a single CPU.

The performance difference between PolyaPeak and other peak callers can be caused by different factors. Even for the relatively well-controlled comparison between PolyaPeak and MACS, we do not have enough information to tell whether peak shape really helps. For this reason, and also because MACS has already been shown by others to have favorable performance, we did not include more peak callers into our comparisons as these comparisons will not produce generalizable principle for future algorithm design. Instead, we performed a well-controlled comparison between PolyaPeak and the reduced PolyaPeak. The only difference is that the reduced PolyaPeak does not use the peak shape information. This comparison is more informative than the comparison between PolyaPeak and the other peak callers since it clearly shows that peak shape information brings improvement. This produces a generalizable principle which in the future may be used together with other established principles (e.g., adjusting for GC content [12]) to continually improve the peak calling algorithms.

PolyaPeak is specifically designed to re-rank the peaks using the shape information. It is important to point out that PolyaPeak does not model the clustering of multiple peaks within a small region, like in GPS and PICS. As a result, PolyaPeak could down rank regions with several closely spaced peaks. Nevertheless we found that overall the improvements overweigh the sacrifices as shown in the real data results.

When modeling a shape, it is often helpful to encourage some smoothness. In our model, this can be achieved by introducing some regularization procedures such as to penalize the second-order derivative of the parameter 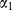 with respect to the bin locations. Such a model could potentially further improve the results, especially when the peaks are wider. This is a research topic worth exploring in the near future.

PolyaPeak is able to improve TFBS detection because there is strong shape information at the true binding sites in TF ChIP-seq experiments. Examples shown in Figure 4 shows that peaks containing large number of reads but didn't show the desired pattern are often down-ranked. On the other hand, peaks containing relatively less reads but showing strong pattern will be up-ranked. ChIP-seq can also be used to study histone modifications (HMs). However, HM ChIP-seq signals usually are highly variable in terms of their widths and shapes. Therefore, a future research topic is to explore whether peak shape is helpful for analyzing HM ChIP-seq data as well.

## Supporting Information

File S1 (PDF)
