# Supplementary material for: PolyaPeak: Detecting Transcription Factor Binding Sites from ChIP-seq Using Peak Shape Information
Source: PLoS One. 2014 Mar 7;9(3):e89694. doi: 10.1371/journal.pone.0089694 (PMC3946423; doi:10.1371/journal.pone.0089694)
Supplement: File S1 — (PDF) [file pone.0089694.s001.pdf]

A A review of MM algorithm for estimating parameters of Pólya distribution

In this section, we briefly review the MM algorithm for fitting multivariate Pólya distributions as developed in Zhou and Lange (2010). Assume there are I independent observations, and each one is a vector of J non-negative integers. Denote the ith observation by $\mathbf{Y}_i = \{Y_{ij} : j = 1, \ldots, J\}$, and denote all data by $\mathbf{Y} = \{\mathbf{Y}_i : i = 1, \ldots, I\}$. Define $T_i = \sum_j Y_{ij}$, and $\mathbf{T} = \{T_i : i = 1, \ldots, I\}$.

If $\mathbf{Y}_i$ follows a Pólya distribution $MP(T_i, \boldsymbol{\alpha})$, where $\boldsymbol{\alpha} = (\alpha_1, \ldots, \alpha_J)$. Then its probability density is

$$\begin{aligned} P(\mathbf{Y}_i|T_i, \boldsymbol{\alpha}) &= \frac{\Gamma(|\boldsymbol{\alpha}|)}{\Gamma(\alpha_1)\cdots\Gamma(\alpha_J)} \int \binom{T_i}{\mathbf{Y}_i} \prod_{j=1}^{J} \theta_j^{Y_{ij}+\alpha_j-1} d\theta_1 \cdots d\theta_J \\ &= \binom{T_i}{\mathbf{Y}_i} \frac{\Gamma(\alpha_1 + Y_{i1})\cdots\Gamma(\alpha_J + Y_{iJ})}{\Gamma(|\boldsymbol{\alpha}| + T_i)} \frac{\Gamma(|\boldsymbol{\alpha}|)}{\Gamma(\alpha_1)\cdots\Gamma(\alpha_J)} \\ &= \binom{T_i}{\mathbf{Y}_i} \frac{\prod_{j=1}^{J} \alpha_j(\alpha_j+1)\cdots(\alpha_j+Y_{ij}-1)}{|\boldsymbol{\alpha}|(|\boldsymbol{\alpha}|+1)\cdots(|\boldsymbol{\alpha}|+T_i-1)} \end{aligned} \tag{A.1}$$

where $|\boldsymbol{\alpha}| = \sum_{j=1}^{J} \alpha_j$.

The joint probability density of the observed data is

$$P(\mathbf{Y}|\mathbf{T}, \boldsymbol{\alpha}) = \prod_{i=1}^{I} \binom{T_i}{\mathbf{Y}_i} \frac{\prod_{j=1}^{J} \alpha_j(\alpha_j+1)\cdots(\alpha_j+Y_{ij}-1)}{|\boldsymbol{\alpha}|(|\boldsymbol{\alpha}|+1)\cdots(|\boldsymbol{\alpha}|+T_i-1)} \tag{A.2}$$

As shown by Zhou and Lange (2010), the maximum likelihood estimate (MLE) of $\boldsymbol{\alpha}$ (or strictly speaking, a local mode of $\boldsymbol{\alpha}$) can be obtained through a Minorization-Maximization (MM) algorithm.

Conceptually, to maximize an objective function $f(\theta)$, an MM algorithm iterates between two steps. In the first step, one uses the current parameter estimate $\theta^{(n)}$ to construct a surrogate function $g(\theta|\theta^{(n)})$ such that $g(\theta|\theta^{(n)})$ minorizes $f(\theta)$, i.e.,

$$\begin{aligned} f(\theta) \geq g(\theta|\theta^{(n)}) \; \forall \, \theta \neq \theta^{(n)} \\ f(\theta^{(n)}) = g(\theta^{(n)}|\theta^{(n)}) \end{aligned} \tag{A.3}$$

In the second step, one finds θ to maximize the surrogate function $g(\theta|\theta^{(n)})$, which gives a new parameter estimate $\theta^{(n+1)}$. Since

$$f(\theta^{(n+1)}) \geq g(\theta^{(n+1)}|\theta^{(n)}) \geq g(\theta^{(n)}|\theta^{(n)}) = f(\theta^{(n)}) \tag{A.4}$$

$f(\theta^{(n)})$ will never decrease as n increases. The algorithm will converge to a stationary point, usually a mode of the objective function.

For fitting the Pólya distribution, the goal is to maximize the log-likelihood function which is written by Zhou and Lange (2010) as:

$$
\begin{aligned}
l(\boldsymbol{\alpha}) &= \log P(\mathbf{Y}|\mathbf{T},\boldsymbol{\alpha}) = -\sum_{c} r_c \log(|\boldsymbol{\alpha}|+c) + \sum_{j=1}^{J}\sum_{c} s_{jc}\log(\alpha_j + c) + constant \\
r_c &= \sum_{i=1}^{I}\delta(T_i \geq c+1),\ s_{jc} = \sum_{i=1}^{I}\delta(Y_{ij} \geq c+1) \qquad \text{(A.5)}
\end{aligned}
$$

Here c ranges from 0 to $\max_i(T_i) - 1$. $\delta(.)$ is an indicator function. $\delta(.) = 1$ if its argument is true, and $\delta(.) = 0$ otherwise.

Using two known inequalities

$$
-\log(c+\alpha) \geq -\log(c+\alpha^{(n)}) - \frac{1}{c+\alpha^{(n)}}(\alpha - \alpha^{(n)}), \qquad \text{(A.6)}
$$

$$
\log(\sum_{j=1}^{J}\alpha_j) \geq \sum_{j=1}^{J}\frac{\alpha_j^{(n)}}{\sum_{j'=1}^{J}\alpha_{j'}^{(n)}}\log(\frac{\sum_{j'=1}^{J}\alpha_{j'}^{(n)}}{\alpha_j^{(n)}}\alpha_j) \qquad \text{(A.7)}
$$

for which the equality holds when $\alpha = \alpha^{(n)}$, one can obtain a surrogate function $g(\boldsymbol{\alpha}|\boldsymbol{\alpha}^{(n)})$ that minorizes $l(\boldsymbol{\alpha})$:

$$
g(\boldsymbol{\alpha}|\boldsymbol{\alpha}^{(n)}) = -\sum_{c} r_c \frac{|\boldsymbol{\alpha}|}{|\boldsymbol{\alpha}^{(n)}|+c} + \sum_{j=1}^{J}\sum_{c} s_{jc}\frac{\alpha_j^{(n)}}{\alpha_j^{(n)}+c}\log(\alpha_j) + constant \qquad \text{(A.8)}
$$

By solving $\partial g(\boldsymbol{\alpha}|\boldsymbol{\alpha}^{(n)})/\partial\alpha_j = 0$, one can obtain the MM update in the nth iteration as:

$$
\alpha_j^{(n+1)} = \left(\sum_{c}\frac{s_{jc}\alpha_j^{(n)}}{\alpha_j^{(n)}+c}\right) \Big/ \left(\sum_{c}\frac{r_c}{|\boldsymbol{\alpha}^{(n)}|+c}\right) \qquad \text{(A.9)}
$$

B Comparison between PolyaPeak and T-PIC

T-PIC analyzes the aligned sequence reads and report detected peaks. However, the software does not rank the peaks. Therefore one cannot directly compare the PolyaPeak and T-PIC in terms of peak ranking. To compare these two algorithms, we used a different approach. For each test dataset, we first run T-PIC with a stringent p-value cutoff (1e-5) and other default parameters to find peaks. We then chose the same number of peaks from the PolyaPeak ranked peak list. The motif enrichment levels of these two peak lists were compared. In principle, one can also run PolyaPeak first and then ask T-PIC to produce the same number of peaks by adjusting the cutoff. However, in T-PIC the p-values are determined using Monte Carlo simulations. The resolution of

the p-values depends on the number of simulations. With reasonably large number of simulations (50,000) T-PIC was already very slow and required hours for a single run. One cannot afford running T-PIC repeatedly by trial and error. For example, if one wants to decrease the peak list size, one will have to use more stringent p-value cutoffs which will significantly increase the computation time. Therefore we used our current strategy in which PolyaPeak was used to match the number of peaks reported by T-PIC. The overall motif content is listed in the table below.

	# peaks	% with motif - PolyaPeak	% with motif - T-PIC
mESC OCT4	4897	29.5	25.3
mESC MYCN	9388	32.1	26.1
K562 MAX	27113	17.8	16.6
K562 MYC	46675	49.5	45.3
HepG2 GABP	71793	6.6	3.9
HepG2 NRSF	58075	1.4	0.6

Our results show that in all datasets, PolyaPeak had higher or comparable percentage of peaks that contain motifs compared to T-PIC. T-PIC reported large numbers of peaks without ranking under the stringent p-value cutoff. We note that in real applications, biologists often wish to pick up the top peaks to do follow-up experiments. Without a ranking, selecting candidates for follow-up studies would be very difficult.

C Effects of the first step peak calling results

Since PolyaPeak works as peak ranker, its performance could be affected by the first step peak calling results. For example, if the first step results have better spatial resolution, the peak shapes can be better estimated which will subsequently improve the results. Throughout the manuscripts, MACS was used as the first step peak caller. To evaluate the effects, we also tried using GPS as the first step peak caller. The figure below shows the motif content comparisons. For clarity, we only compared the results from MACS, GPS and PolyaPeak with MACS/GPS as the first step peak caller (labeled as MACS+PolyaPeak and GPS+PolyaPeak). It shows that PolyaPeak usually provide better rankings overall, that is, line 1 is higher than line 2 and line 3 is higher than line 4 in most datasets. These results demonstrate the robustness of PolyaPeak.

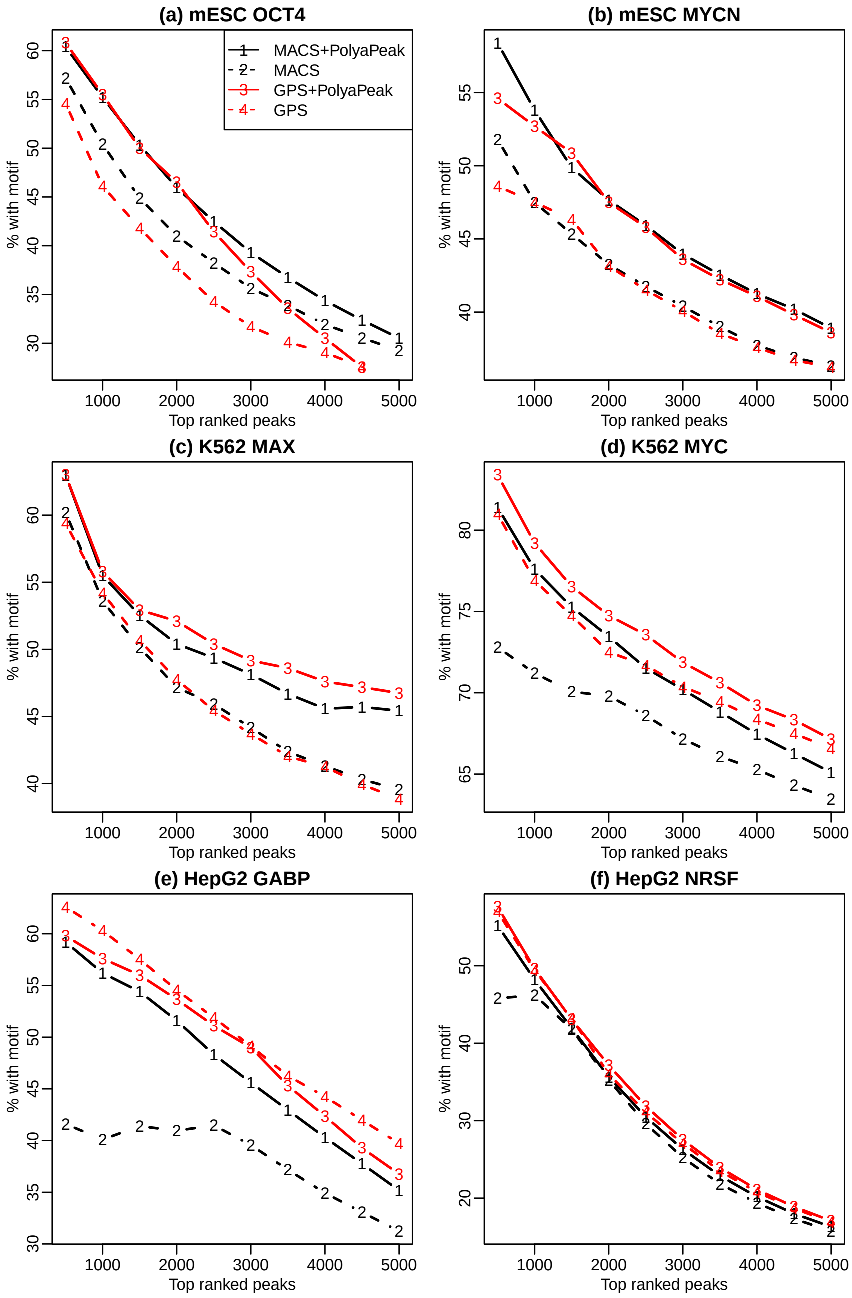

References

[1] Zhou H, Lange K: **MM algorithms for some discrete multivariate distributions**. *Journal of Computational and Graphical Statistics* 2010, **19**(3):645–665.
